# Relationship between Fertility Traits and Kinematics in Clusters of Boar Ejaculates

**DOI:** 10.3390/biology10070595

**Published:** 2021-06-28

**Authors:** Vinicio Barquero, Eduardo R. S. Roldan, Carles Soler, Bernardo Vargas-Leitón, Francisco Sevilla, Marlen Camacho, Anthony Valverde

**Affiliations:** 1 Animal Reproduction Laboratory, San Carlos Campus, Costa Rica Institute of Technology, School of Agronomy, Alajuela 223-21002, Costa Rica; vinicio1196@gmail.com (V.B.); fsevillabenavides@gmail.com (F.S.); mcamacho@itcr.ac.cr (M.C.); 2 Department of Biodiversity and Evolutionary Biology, Museo Nacional de Ciencias Naturales (CSIC), 28006 Madrid, Spain; roldane@mncn.csic.es; 3 Department of Cellular Biology, Functional Biology and Physical Anthropology, University of Valencia, Campus Burjassot, C/Dr Moliner, 50, 46100 Burjassot, Spain; carles.soler@uv.es; 4 Population Medicine Research Program, Veterinary Medicine School, National University of Costa Rica, Heredia 40104, Costa Rica; bernardo.vargas.leiton@una.cr

**Keywords:** sperm, cluster, sows, motility, CASA-Mot, artificial insemination

## Abstract

**Simple Summary:**

Swine reproduction efficiency is determined by the fertility potential of the sow and sperm quality. The objective of this study is to compare boar sperm motility and kinematic features to evaluate their relationships with reproductive success after artificial insemination (AI). In this study, the movement patterns of boar ejaculates were analyzed by a computer-assisted semen analysis (CASA)-Mot system, and the kinematic values of ejaculate clusters were assessed. The semen of the Pietrain boars showed more linear trajectory of the spermatozoa, while curvilinear velocity and oscillatory movement characterized the semen of the Duroc × Pietrain boars. The offspring of sows inseminated with Pietrain boars showed significantly lower number of stillbirths. In addition, ejaculate grouping into clusters did not have a predictive capacity on litter size variables. Nevertheless, the kinematic variables of the ejaculate may have a predictive, albeit reduced, capacity regarding litter size variables. The results of this study therefore open up possibilities for future assessments of fertility.

**Abstract:**

The aim was to determine the relationship between kinematic parameters of boar spermatozoa and fertility rates of sow, as well as to assess the effect of sperm clusters on the fertility capacity of the ejaculate. Semen samples were collected from 11 sexually mature boars. Samples were analyzed by an ISAS^®^v1 CASA-Mot system for eight kinematic parameters. Ejaculate clusters were characterized using multivariate procedures, such as principal factors (PFs) analysis and clustering methods (the k-means model). Four different ejaculate clusters were identified from two kinematic PFs which involved linear trajectory and velocity. There were differences (*p* < 0.05) between the sperm kinematic variables by sire line. There was no statistical difference (*p* > 0.05) between dam lines and ejaculate clusters in fertility variables. The discriminant ability of the different kinematics of sperm variables to predict litter size fertility was analyzed using receiver operating characteristics (ROC) curve analysis. Curvilinear velocity (VCL), average path velocity (VAP), amplitude of lateral head displacement (ALH), and beat-cross frequency (BCF) showed significant, albeit limited, predictive capacity for litter size fertility variables (range: 0.55–0.58 area under curve, AUC). The kinematic analysis of the ejaculates in clusters did not have a predictive capacity for litter size variables.

## 1. Introduction

Several countries have implemented frequent artificial insemination (AI) in pigs [1,2,3], which has been associated with increased litter size [4,5,6]. AI increases the rate of genetic progress because it is designed to capture the advantages of heterosis; it increases the feed efficiency, growth, and litter size while also reducing problems related to boars [7]. One of the most important advantages of AI is that it requires no relocation of the sire, and a single ejaculate can be used to inseminate 10 to 20 sows [8]. The semen sample should be diluted and preserved at 17 °C [9] in order to extend useful sperm life, durability, and quality [10,11,12].

The sperm quality needs to be evaluated to understand the fertility and genetic value of the boar. Because of this, boars are housed in an artificial insemination station to optimize management, health, and fertility [8,13]. Computer-assisted semen analysis (CASA) has been used to evaluate the motility and kinematic parameters of the ejaculate, and more than 70% total motility must be achieved for the ejaculate to be considered for insemination of sows [14]. CASA systems have a pre-determined set-up to assess some species [15,16], boars included, but configurations must be validated to assure the precision and accuracy of the results.

Motility evaluation is based on kinematic patterns [16,17]. Sperm motion is obtained by capturing consecutive frames of a posterior structuration of the trajectory [17,18,19]. CASA-Mot systems provide accuracy in the results of motility and kinematic parameters, compared with the subjective methods used in the past [15]. However, this analysis provides large volumes of data [20] that must be evaluated using multivariate statistics [21] to reduce the dimensionality of the kinematic variables. Furthermore, for accuracy in the assessment, some factors must be considered, such as software [22], capture fields [23], recording time [24], counting chambers [25,26], and frame rate [27,28,29]. Therefore, to reduce the variance of the results, a 20 μm depth for counting chambers in boars has been recommended because it provides adequate space to promote the movement of the spermatozoa [23,30].

Several authors have indicated that the ejaculate is composed of a heterogeneous population of spermatozoa according to their kinematic variables [31,32,33,34]. Sperm subpopulations have been described in many species [20,26,29,35,36,37,38,39]; nevertheless, the biological meaning in terms of the physiological functions associated with fertility is still being studied [40]. Even today, there are multiple clustering approaches that can be used to estimate sperm subpopulations in the ejaculate, and different procedures are analyzed to determine the statistical and biological relevance of these subpopulations [41,42]. This paper characterized ejaculates into clusters for description. This approach was based on the idea that we do not know which spermatozoa fertilized the sow, and which sperm subpopulation contained that cell.

The principal parameters to determine sow fertility are litter size (LS), piglets born alive [8,43], stillbirth or mummified piglets [8], and farrowing or conception rate [44,45,46,47]. Several studies have shown that sow fertility has a positive relationship with some kinematic parameters of boar sperm, such as curvilinear velocity (VCL), straight line velocity (VSL), and beat cross frequency (BCF) [8,43,48,49,50]. The aim of the present study was to determine the relationship between kinematic parameters of boar spermatozoa and fertility rates of multiparous sows, using a commercial CASA-Mot system, as well as to determine the effect of sperm clusters on the fertility capacity of the ejaculate.

## 2. Materials and Methods

### 2.1. Animals

The experiment was conducted at a commercial swine farm (Agropecuaria Los Sagitarios S.A., Alajuela, Costa Rica) in 2019–2020 in the northwest of Costa Rica (Río Cuarto, 10°20′32″ N, 84°12′55″ W, Alajuela, Costa Rica, Central America), following the laws and regulations for experiments on live animals in Costa Rica. This study was performed following ethical principles, and with the approval of the Committee of Centro de Investigación y Desarrollo de la Agricultura Sostenible para el Trópico Húmedo at the Costa Rica Institute of Technology (CIDASTH-ITCR), according to Section 08/2020, article 1.0, DAGSC-100-2020.

Eleven sexually mature and healthy boars from two commercial terminal sire lines (SL: Duroc × Pietrain (*n* = 8) and Pietrain boars (*n* = 3)), 23.3 ± 8.5 months of age at the beginning of the experiment and with known fertility, were used as semen donors in this study. For the study, breeding boars were housed individually in well-ventilated pens with average temperature of 25.60 ± 2.94 °C during the time of the experiment. Ejaculates were collected in rainy season. Females came from four crossbred dam genetic lines (DL: York (Y), Landrace (L) and Pietrain (P); with the following crossing schemes YLP-50 (¼ Y × ¼ L × ½ P), YLP-75 (^1^/_8_ Y × ^1^/_8_ L × ^3^/_4_ P), YLP-87.5 (^1^/_16_ Y × ^1^/_16_ L × ^7^/_8_ P); and Y-L-50 (½ Y × ½ L)). All the females were bred at the farm and they came from within maternal crossing schemes such as the continuous 3-generation cross between YLP hybrid sows and P boars. Mean sow parity for all dam genetic lines was 4.10 ± 2.76. The animals were fed with the standard breeder mixture (made on the farm), containing maize, soybean meal, mineral mixture, and common salt, to fulfill their nutrient requirements [51]. Concentrate was provided to pregnant sows—2.5 kg in the first 2/3 of gestation and 3 kg in the final third—and males consumed 2.5 kg per day and were provided with water ad libitum.

### 2.2. Fertility Trial

A total of 816 triple artificial inseminations performed with homospermic ejaculates were evaluated in 272 sows. These AIs were conducted randomly with 40 ejaculates from 11 males. Each genetic line of females was randomly inseminated with each genetic line of males. The ejaculates were used within 3 days of collection, and only those used to inseminate at least three females were evaluated. The mean number of inseminated sows per boar was 24.7 ± 10.1 females. All crossbred dam lines were inseminated with seminal doses from each sire line. Fertility rate was measured as females pregnant/total number of females inseminated (%). Total piglets born per litter (TPB), piglets born alive (PBA), stillbirth (SB), number of mummies (MP), and litter weight (LW; kg) were used as fertility variables, and those parameters were measured at the farrowing time.

### 2.3. Collection and Examination of Semen

Semen samples were collected in the morning, once per week, using the “gloved-hand” technique [52] and immediately placed in a water bath at 37 °C in the farm laboratory. In all cases, the sperm-rich fractions were collected and diluted 1:1 (vol:vol) by one-step with a commercial extender (Zoosperm ND5; Import-Vet, Barcelona, Spain). Insemination doses contained 3.7 ± 1.3 × 10^9^ spermatozoa. From each boar, 3.64 ± 0.81 ejaculates were obtained. Samples from each ejaculate were evaluated for motility, and only ejaculates with at least 70% motile spermatozoa and 85% morphologically normal spermatozoa were used. The concentration was measured with Spermacue (Minitube, GmbH, Tiefenbach, Germany) following established protocols [24]. Samples were stored at 17 °C and were then transported to the laboratory in the same refrigerated conditions (17 °C) used for commercial distribution. A volume of one milliliter (1 mL) of mixed samples was placed in an Eppendorf^®^ tube (Sigma-Aldrich, St. Louis, MO, USA) and maintained at 37 °C for 30 min before use.

### 2.4. Assessment of Sperm Variables

For the analysis of motility, ISAS^®^ D4C20 disposable counting chambers (Proiser R+D., Paterna Spain) were used after being pre-warmed at 37 °C. After thorough mixing of the diluted semen samples, a volume of 2.7 µL was distributed along the counting chamber race by capillarity to fill it completely. Analyses were conducted using the CASA-Mot system ISAS^®^v1 (Integrated Semen Analysis System, Proiser R+D, Paterna, Spain) fitted with a video-camera (Proiser 782M, Proiser R+D), with 25 frames acquired per field at a frame rate of 50 Hz and final resolution of 768 × 576 pixels. The camera was attached to a microscope UB203 (UOP/Proiser R+D) with a 1× eyepiece and a 10× negative phase contrast objective (AN 0.25), and an integrated heated stage maintained at a constant temperature of 37.0 ± 0.5 °C. The CASA settings used were a particle area between 10 and 80 μm^2^ and connectivity of 11 μm. The percentage of total motile cells and progressive motility (%) corresponded to spermatozoa swimming forward quickly in a straight line. The following parameters defined progressive motility: straightness (STR, straightness index) ≥45% and average path velocity (VAP) ≥25 µm·s^−1^, defined as the average velocity over the smoothed cell path. A single technician carried out the assessments of sperm morphology. Sperm were classified as having normal or abnormal morphologic features following WHO strict criteria [53]. A total of 200 sperm were analyzed per slide; 100 sperm from each of two different locations on the slide were assessed. If the difference between the percentage of normal sperm in the two areas was 5% or less, then the mean value was calculated.

### 2.5. Computerized Kinematics Analysis

The CASA analyses were performed in seven microscope fields on a total of at least 600 cells per sample. The CASA-Mot variables assessed in this study included straight-line velocity (VSL, µm·s^−1^), corresponding to the straight line from the beginning to the end of the track; curvilinear velocity (VCL, µm·s^−1^), measured over the actual point-to-point track followed by the cell; average path velocity (VAP, µm·s^−1^), the average velocity over the smoothed cell path; amplitude of lateral head displacement (ALH, µm), defined as the maximum of the measured width of the head oscillation as the sperm swims; beat-cross frequency (BCF, Hz), defined as the frequency with which the actual track crosses the smoothed track in either direction; motility (%), defined as the percentage of total motile cells; and progressive motility (%), corresponding to spermatozoa swimming rapidly forward in a straight line. Three progression ratios, expressed as percentages, were calculated from the velocity measurements described above: linearity of forward progression (LIN = VSL/VCL·100), straightness (STR = VSL/VAP·100), and wobble (WOB = VAP/VCL·100).

### 2.6. Statistical Analysis

The data obtained from the evaluations of all ejaculates and fertility were analyzed by descriptive statistics. Distribution properties for all variables were also explored using histograms and probability plots.

#### 2.6.1. Multivariate Procedures

A subset of data was created with the means per ejaculate of all eight kinematic variables. Multivariate procedures were performed to identify ejaculate clusters from this subset of sperm kinematic data. This approach was founded on the fact that we do not know which spermatozoa fertilized the sow, and which sperm subpopulation contained that cell. All the values for the kinematic variables were standardized to avoid any scale effect. A principal factor analysis (PFA) was performed on these data to derive a small number of linear combinations that still retained as much information as possible from the original variables. Prior communalities for this analysis were estimated from the maximum absolute correlation coefficient between each variable and any other. The number of principal factors (PF) to be extracted was determined from the Kaiser criterion, namely by selecting only those with an eigenvalue >1. The KMO (Kaiser-Meyer-Olkin) statistic was also obtained [21] as a measure of dataset adequacy for factor extraction. As a rotation method, the varimax method with Kaiser normalization was used [54]. Correlations between factors and original kinematic variables were explored to better understand the meaning of the factors extracted.

Further, an analysis was conducted to classify the ejaculates into a reduced number of clusters, based on scores obtained from factor analysis. This was accomplished in two phases, combining hierarchical and non-hierarchical clustering procedures. First, factor scores for all ejaculates were clustered hierarchically using the Ward Minimum Variance method [55]. From this analysis, an optimal number of clusters was determined based on criteria such as the Cubic Clustering Criterion (CCC), Pseudo-T, Pseudo-F, and partial R^2^. Second, the optimal number of clusters obtained in the previous analysis was used as the target number of clusters in a non-hierarchical K-means cluster analysis [56].

ANOVA was further applied to evaluate statistical differences between clusters for all kinematic variables. The threshold for significance was defined as *p* < 0.05. Further, pairwise comparison between cluster means were performed by the Tukey-Kramer test. Results were presented as mean ± standard deviation of the mean. All data were analyzed using the SAS 9.4 [57] statistical program.

#### 2.6.2. GLMM Model on Sow Fertility Parameters

Sow fertility variables were analyzed using the Generalized Linear Mixed Models (GLMM). The response variables were litter weight, litter size, piglets born alive, stillbirth, and number of mummies. A normal distribution with an identity link function was assumed for litter weight, while a Poisson distribution with a log identity link function was assumed for all other response variables. Ejaculate clusters, obtained from multivariate analysis, was considered as the main fixed independent factor in the model. Other fixed factors with potential effects on sow fertility were also added to the model, such as dam line, sire line, dam × sire line interaction, nested boar within sire line, month of farrowing, pregnancy length, different parities, and length of period between previous and present ejaculate. A random residual effect was also added to the model to account for correlations between different ejaculates obtained from the same boar. GLMM analysis was performed with the SAS 9.4 [57] statistical program.

#### 2.6.3. ROC Analysis

The diagnostic test with a dichotomous outcome (positive/negative fertility test results) of the different kinematic semen variables to predict litter size fertility was analyzed using receiver operating characteristic (ROC) curve analysis. The approach of diagnostic test evaluation uses sensitivity and specificity as measures of accuracy of the test, in comparison with standard status (farrowing). The sensitivity (true positive rate) and specificity (true negative rate) of each kinematic variable vary across the different thresholds, and the sensitivity was inversely related with specificity. The plot of sensitivity versus 1-Specifity is called receiver operating characteristic (ROC) curve and the area under the curve (AUC). AUC varies from 0.5 (test with no discriminatory ability) to 1 (perfect discriminatory ability). An ROC was also used to calculate the elective breaking point (cut-off value) for each kinematic sperm variable. The analysis may also be used to determine the optimal cut-off value (optimal decision threshold).

## 3. Results

### 3.1. Descriptive Analysis of Semen Evaluation

Sperm concentration, volume of semen, and total spermatozoa in the ejaculate were 374.23 ± 129.24 × 10^6^/mL, 231.98 ± 63.08 mL and 82.04 ± 23.73 × 10^9^, respectively. The sperm concentration (million/mL) was 378.63 ± 134.98 in the Duroc × Pietrain crossbred and 361.00 ± 112.35 in the Pietrain. Total motility (%) of boar samples was 77.36 ± 11.17, with an overall range of 35.05–93.69%. The progressive motility of sperm (%) was 63.76 ± 11.96. Average total motility (%) for Duroc × Pietrain and Pietrain boars was 81.28 ± 7.76 and 65.61 ± 11.73, respectively (*p* < 0.05). The progressive motility (%) was 67.00 ± 10.05 (Duroc × Pietrain) and 54.04 ± 12.19 (Pietrain) (*p* < 0.05).

### 3.2. Analysis of the Ejaculate Cluster Structure

**Figure 1:**
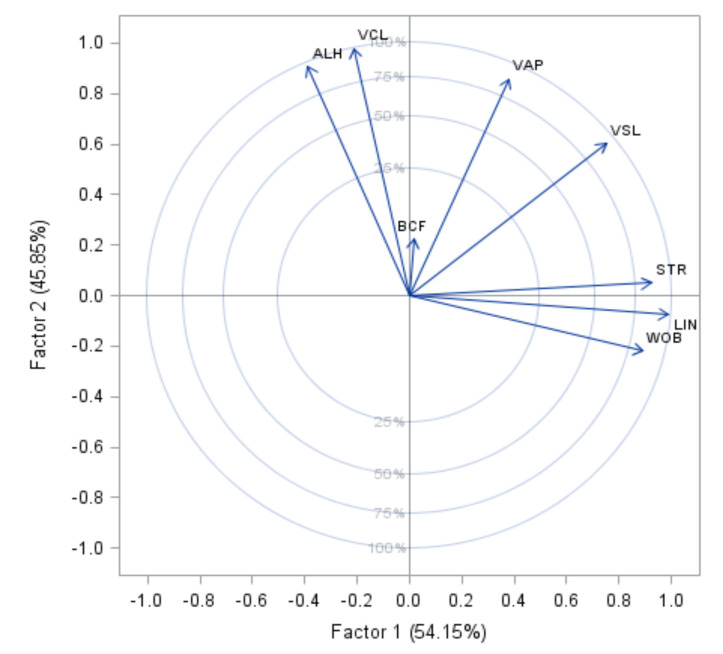
Distribution of factor loading of kinematic variables for boar spermatozoa on the plane conformed by two first principal factors (PFs with % variance explained). VCL: curvilinear velocity; VSL: straight-line velocity; VAP: average path velocity; LIN: linearity of forward progression; STR: straightness; WOB: wobble; ALH: amplitude of lateral head displacement; BCF: beat-cross frequency.

Principal factors analysis indicated a KMO statistic of 0.56, and final communality estimates were above 0.85 for all kinematic variables, except BCF (0.05). According to Kaiser criterion, two significant PF can be extracted from these data, both accounting for 98% of the total variance. The first PF, defined as linear trajectory (PF1), was responsible for 53% of the variance and was mainly associated with the kinematic variables LIN, STR, WOB, and VSL, with the largest correlation being for LIN (0.99). The second PF, defined as velocity (PF2), was strongly associated with the variables VCL, VAP, VSL, and ALH, with the largest correlation being for VCL (0.98) (Table 1). This factor also indicated that ejaculates whose sperm presented a linear trajectory had a relatively greater effect on the total variance than the ejaculates where sperm velocity was faster (Figure 1).

**Figure 2:**
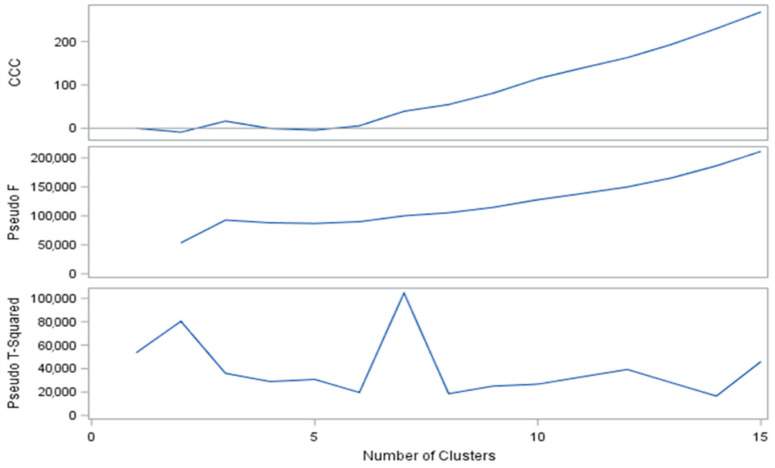
Optimal number of groups (ejaculate clusters) based on the statistical criteria of Cubic Clustering Criterion (CCC), Pseudo-T, Pseudo-F, and partial R^2^.

**Figure 3:**
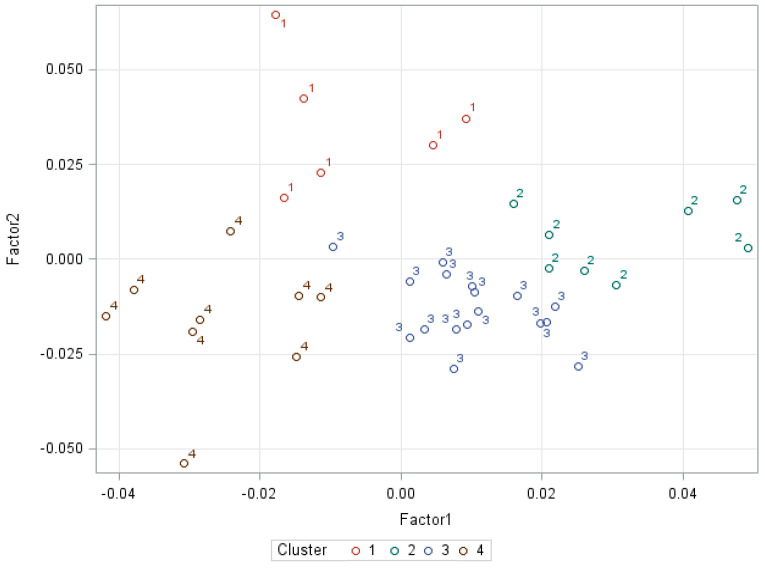
Distribution of ejaculates according to the principal factors value and prediction ellipse of the clusters (sperm populations).

The optimal clustering level is obtained when local peaks of CCC, a high value of Pseudo F, and a low value of Pseudo T2 are combined together with a high value (Figure 2). This was approximately at group level 3; however, level 4 could be better by group location. The stability and accuracy of the grouping by cluster was contrasted by the coefficient of determination (R^2^ = 0.75). The model was adjusted in the cluster procedures analysis with R^2^ in each repetition for better validation (Figure 3).

Boar ejaculates were grouped into four clusters according to hierarchical Ward’s minimum variance, followed by non-hierarchical k-means clustering procedures (Table 2). The kinematic parameters characterized the sperm movement in the ejaculate clusters (ECs). Cluster 1 (EC1) contained the sperm with the highest VCL and VAP (92.08 ± 5.12 µm·s^−1^; 51.79 ± 3.28 µm·s^−1^ respectively). These ejaculates present a spermatozoa with highest BCF and ALH (8.93 ± 0.59 Hz; 3.46 ± 0.23 µm respectively). Cluster 2 (EC2) included sperm characterized by high VSL (42.91 ± 2.76 µm·s^−1^), and the highest values of LIN and STR (57.17 ± 3.59%; 82.34 ± 1.50% respectively). Cluster 3 (EC3) contained the ejaculates whose spermatozoa had a high oscillatory movement, indicated by WOB and BCF (63.72 ± 2.86%; 8.73 ± 0.52 Hz respectively), and an intermediate value of STR (75.72 ± 5.58%). Cluster 4 (EC4) exhibited ejaculates whose sperm were less linear and progressive, as indicated by the lowest VSL and VAP (24.00 ± 4.68 µm·s^−1^; 37.21 ± 5.56 µm·s^−1^ respectively), together with the lowest values of LIN and STR (37.26 ± 5.37%; 65.48 ± 6.66% respectively).

### 3.3. Relationship between Kinematics Cluster and Fertility

The mean fertility rate was 69.60 ± 21.67%. There were no differences between sire lines for this variable. There were differences (*p* < 0.05) between the sperm kinematic variables by sire line. The kinematic variables of semen of the Pietrain boars showed more linear trajectories of the spermatozoa, whereas semen from Duroc × Pietrain boars were characterized by curvilinear velocity and oscillatory movement of the sperm (Table 3). Fertility results based on the mean values of each cluster indicate differences (*p* < 0.05) between ejaculate clusters for total piglets born per litter (TPB), stillbirth (SB), and litter weight (LW; kg). There were no significant differences between ejaculate clusters (*p* > 0.05) for piglets born alive (PBA) and number of mummies (MP). However, some trends were observed. EC3 had higher PBA (10.25 ± 1.32), but higher MP (0.32 ± 0.17); moreover, LW was also higher (18.83 ± 1.71 kg). EC2 was characterized by intermedium fertility rates in all categories, exhibiting a higher value in PBA (9.55 ± 1.28) than EC1 and EC4. Finally, EC4 had the lightest litter (11.47 ± 2.03 kg); because of this, EC4 presents the lowest TPB (7.64 ± 1.13), and also reported the lowest SB (0.12 ± 0.07) and intermedium MP (0.19 ± 0.14). The fertility variables characterized according to ECs indicate that EC3 showed the highest litter size, while EC4 presented the lowest value of total piglets born per litter. EC2 and EC3 presented higher values of stillbirth than EC1 and EC4 (Table 4). The fertility variables characterized according to sire genetic line did not show differences (*p* > 0.05), except for significantly fewer stillbirths in Pietrain boars (Table 5).

### 3.4. Fertility Variables by Dam and Sire Genetic Line

**Figure 4:**
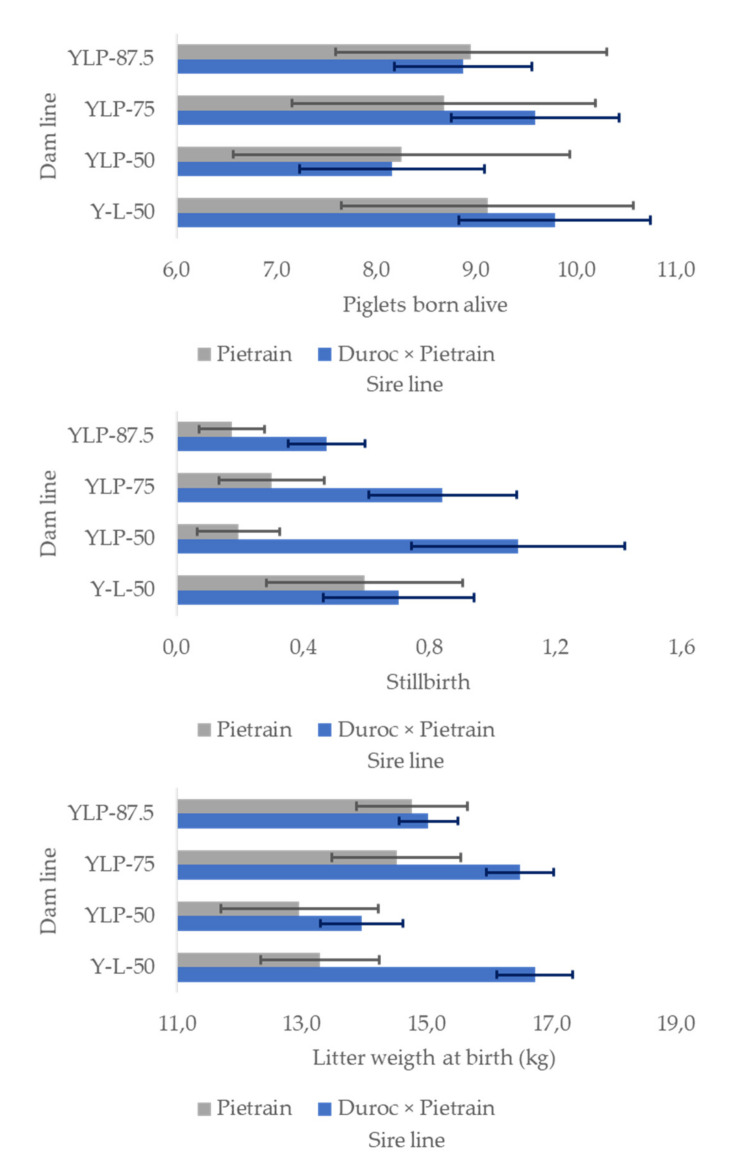
Dam genetic line effect on the values of piglets born alive, stillbirth, and litter weight at birth (kg). Y: York, L: Landrace, P: Pietrain, YLP-50 = (¼ Y × ¼ L × ½ P), YLP-75 = (^1^/_8_ Y × ^1^/_8_ L × ^3^/_4_ P), YLP-87.5 = (^1^/_16_ Y × ^1^/_16_ L × ^7^/_8_ P), Y-L-50: ^1^/_2_ Y × ^1^/_2_ L. *p* < 0.05.

Fertility parameters did not show significant differences (*p* > 0.05) between dam lines. The hybrid (Y-L-50) presents the highest value in TPB (10.44 ± 0.96), PBA (9.45 ± 0.92), and SB (0.65 ± 0.21). YLP-87.5 showed the lowest MP (0.13 ± 0.08), followed by Y-L-50 (0.15 ± 0.11). The heaviest litter was for YLP-75 (15.53 ± 1.32 kg), which represents 103.22% of the value of Y-L-50 (Figure 4). The Pietrain sire line had fewer SBs than Duroc × Pietrain (0.74 ± 0.16), but all the other parameters showed no differences (*p* > 0.05). Pietrain had lower values of MP (0.13 ± 0.09), but Duroc × Pietrain presented a higher value of TPB (10.27 ± 0.65) and PBA (9.08 ± 0.61) (Figure 4).

### 3.5. Predictive Capacity of Fertility

The sperm kinematic variables with significant results in the ROC curve analysis are presented in Table 6. VCL, VAP, ALH, and BCF showed significant, albeit limited, predictive capacity for litter size fertility variables (range: 0.55–0.58 AUC). Cut-off values, with their sensitivities and specificities, are also presented in Table 6. The best cut-off points to identify ejaculates with low fertility potential in relation to number of mummies were 70.70 µm·s^−1^ VCL, 42.40 µm·s^−1^ VAP, and 2.59 µm ALH. Similarly, ejaculate clusters showed limited predictive capacity for litter size variables (data not shown).

## 4. Discussion

The number of sows inseminated by a single ejaculate depends on the concentration and quality of the semen [46]. This quality is measured by microscopic analysis of the samples, which allows maximization of the number of doses per boar [23]. The selection criteria of the genetic line or breed must be taken in the context of the farm’s purpose [58] and the semen quality [59], because the fertility of the sows is related to semen characteristics, such as kinematic variables [8,43,48,49,50], morphometric values [60], DNA fragmentation [61], concentration [62], and viability [63]. Our results indicate a mean concentration of 3.7 × 10^9^ spermatozoa per AI dose. Our multivariate analysis found four ejaculate clusters, where EC1 was characterized by rapid and progressive spermatozoa; this cluster had only ejaculates from Duroc × Pietrain boars. Moreover, EC4 was mainly ejaculates provided by Duroc × Pietrain boars, and was characterized by slow and non-progressive sperm. The major proportion of Pietrain ejaculates was in EC3; this cluster had ejaculates with slow and progressive spermatozoa. In EC2, both sire lines were better distributed, and the ejaculates were characterized by medium velocity and linear progressively motile spermatozoa.

Some authors have found that sperm parameters, such as motile spermatozoa, do not predict litter size [64,65]. Other studies have found similar results with respect to the effect of motile spermatozoa on fertility of litter size, indicating that when total motile spermatozoa is more than 60% and there is a concentration of 3 × 10^9^ spermatozoa per AI dose, no relationship with fertility parameters is found [66,67]. This study showed that Duroc × Pietrain boars have better patterns in motile and progressively motile spermatozoa than Pietrain boars. These data could indicate that Duroc × Pietrain boars have the best fertility data, and the results of TPB showed that Duroc × Pietrain had the highest value, and that the value was relevant. Several authors have asserted that the kinematic values are related to fertility data of the sows, such as litter size [8,43,48], pregnancy rate [49,50], and fertility index [50]. The most related variables of kinematics are VSL [8,48], VCL [8,43,50], and BCF [8,49,50]. Our results showed that EC1 had the highest values of VCL and BCF, and an intermediary value of VSL. Multivariate procedures showed that the sire line had no effect (*p* > 0.05) on the fertility data. Non-relevance of the difference between boar racial groups could be due to the fact that the clusters are very close in Euclidean distance with respect to the centroids, and some ejaculates were assigned to a specific group (cluster) but maintained some motile spermatozoa and/or kinetic patterns similar to ejaculates from another cluster. The main obstacle to estimating the fertility of the boar is that it is necessary for a large number of sows to be inseminated over a long period of time, requiring an extended time period for the study, and during this time the boar fertility could change [65]. In our study, we determined that the coefficient of variation in motile spermatozoa ranged from 1.48% to 24.26%, while the percentage of progressively motile spermatozoa ranged from 5.41% to 37.69%. Differences between boars could be due to individual variability, breed, and age [8,49,68]. The ejaculates in the present study showed within and between boar differences. Furthermore, we characterize the assessed ejaculates on the basis of variables such as sperm velocity and progressiveness, being able to describe both rapid and progressive ejaculates as well as slow and non-progressive ejaculates. In our study, we characterized ejaculates into clusters for description. Other studies have described subpopulations of spermatozoa [26,29,33,34,36,37,38,39,69,70,71]. However, we believed it more appropriate to regard them as ejaculates because we do not know which spermatozoa fertilized the sow, and which sperm subpopulation contained that cell. Fertility is multifactorial, with semen having an effect only in the final result; on the other hand, the sow has many more influencing factors. Among the main factors that influence sow fertility are weather [72], housing conditions [73], nutritional status [72], duration of gestation [74], endocrine activity [72], and sow lifetime productivity [75]. In the present study, four maternal crosses were considered because maternal ability can influence phenotypic performance in species with numerous litters, such as pigs [76,77,78]. In the case of boars, two racial groups were used because of the selection objective in terminal crossings [79,80,81]. The results indicated that the sows of line Y-L-50 inseminated with Duroc × Pietrain boars presented better litter size yields; this is explained by the genetic potential provided by heterosis and complementarity [82]. There was no difference (*p* > 0.05) between dam lines in the fertility variables, even though those values showed highly variable percentages compared to Y-L-50. However, we suggest that there is biological and economic importance because of the genetic improvement achieved by selecting sows and boars to increase the number of live born piglets and the survival proportion [83]. The prenatal survival of pigs is of great importance [84], and this is linked to the quality of the oocyte [72], which is influenced by the nutrition of the sow [75]. To explain the relevance of the differences between ejaculate clusters, it is necessary to analyze the data; YLP-87.5 has the lowest values of SB and MP, therefore the value of PBA is intermediate even when it has a low TPB. On the other hand, Y-L-50 is the dam line with the highest TPB value, but it is also the one with the most SB, and therefore its PBA quantity is diminished. This study determined the probability of relevance from the Bayesian marginal posterior distribution to confront these findings with the significance *p* < 0.05 from each cluster of boar ejaculates, and in some cases indicated that differences between cluster could be considered irrelevant. Other studies carried out in other species, such as cattle, have described results that indicate that the differences between sperm subpopulations are not relevant [42]. On the other hand, the fertility variables showed differences between EC2 to EC4, which could be explained by the values of the sperm velocity variables and the velocity relationships. In this sense, EC2 presented the highest values of VSL, VAP, LIN, STR, and WOB, while EC4 was the cluster with the lowest values for the same variables. These results indicate the need to continue studying the cluster structure of pig ejaculates, and how these intervene in the fertility functionality of females.

## 5. Conclusions

We have shown that kinematic analysis of boar ejaculates reveals kinematically separate populations. There were differences between the sperm kinematic variables by sire line. However, there was no overall significant difference between dam lines assessed by multivariate procedures. The fertility variables characterized according to the sire genetic line did not show differences, except for significantly fewer stillbirths in Pietrain boars. Sperm kinematic variables may have a predictive capacity for litter size variables, albeit a limited one. Nevertheless, the analysis of the ejaculates into clusters did not have a predictive capacity for litter size variables.

## Figures and Tables

**Table 1:** Correlations between boar sperm kinematic variables (unrotated solution) and latent factors (PF1, PF2) *.

Variable	PF1	PF2
LIN	0.99	
STR	0.92	
WOB	0.89	
VSL	0.75	0.60
VCL		0.98
ALH		0.91
VAP		0.86
BCF		
**Var Exp (%)**	**53.1**	**44.9**

Var Exp: variance explained in each PF. Total variance explained: 98.0%. * Expresses the more important variables in each PF. Only eigenvectors >0.6 are presented. VCL: curvilinear velocity; VSL: straight-line velocity; VAP: average path velocity; LIN: linearity of forward progression; STR: straightness; WOB: wobble; ALH: amplitude of lateral head displacement; BCF: beat-cross frequency. Bold is recommended for the appreciation of the total variance explained.

**Table 2:** Kinematic variables (mean ± SD) of the four ejaculate clusters (ECs).

Variable	EC1	EC2	EC3	EC4
VCL	92.08 ± 5.12 ^a^	75.19 ± 4.54 ^b^	65.48 ± 6.07 ^c^	68.57 ± 9.98 ^b,c^
VSL	38.09 ± 6.52 ^a^	42.91 ± 2.76 ^a^	31.69 ± 3.48 ^b^	24.00 ± 4.68 ^c^
VAP	51.79 ± 3.28 ^a^	50.16 ± 3.43 ^a^	41.30 ± 3.43 ^b^	37.21 ± 5.56 ^b^
LIN	42.23 ± 6.68 ^c^	57.17 ± 3.59 ^a^	49.53 ± 4.49 ^b^	37.26 ± 5.37 ^c^
STR	71.44 ± 7.51 ^b,c^	82.34 ± 1.50 ^a^	75.72 ± 5.58 ^b^	65.48 ± 6.66 ^c^
WOB	57.49 ± 4.54 ^b^	67.35 ± 3.61 ^a^	63.72 ± 2.86 ^a^	55.45 ± 3.75 ^b^
ALH	3.46 ± 0.23 ^a^	2.73 ± 0.17 ^b^	2.47 ± 0.20 ^b^	2.70 ± 0.35 ^b^
BCF	8.93 ± 0.59	8.36 ± 0.43	8.73 ± 0.52	8.44 ± 0.87

EC1: rapid, progressive and undulatory; EC2: medium velocity and linear progressive; EC3: slow, progressive and undulatory; EC4: slow velocity, nonlinear and non-progressive. Number of ejaculates = 40. VCL: curvilinear velocity (µm·s^−1^); VSL: straight line velocity (µm·s^−1^); VAP: average path velocity (µm·s^−1^); LIN: linearity of forward progression (%); STR: straightness (%); WOB: wobble (%); ALH: amplitude of lateral head displacement (µm); BCF: beat-cross frequency (Hz). SD: standard deviation. ^a–c^ Different letters indicate differences between ejaculate clusters. *p* < 0.05.

**Table 3:** Kinematic variables (mean ± SEM) of boar ejaculates by sire genetic line.

	Sire Line	
Variable	Pietrain	Duroc × Pietrain
VCL	71.10 ± 0.18 ^a^	76.15 ± 0.06 ^b^
VSL	39.32 ± 0.11 ^a^	32.25 ± 0.04 ^b^
VAP	47.51 ± 0.11 ^a^	43.70 ± 0.04 ^b^
LIN	55.34 ± 0.14 ^a^	43.68 ± 0.05 ^b^
STR	80.03 ± 0.12 ^a^	72.02 ± 0.04 ^b^
WOB	67.58 ± 0.08 ^a^	58.57 ± 0.03 ^b^
ALH	2.61 ± 0.01 ^a^	2.93 ± 0.02 ^b^
BCF	8.16 ± 0.01 ^a^	8.63 ± 0.02 ^b^

Number of ejaculates = 40. VCL: curvilinear velocity (µm·s^−1^); VSL: straight line velocity (µm·s^−1^); VAP: average path velocity (µm·s^−1^); LIN: linearity of forward progression (%); STR: straightness (%); WOB: wobble (%); ALH: amplitude of lateral head displacement (µm); BCF: beat-cross frequency (Hz). SEM: standard error of the mean. ^a–b^ Different letters indicate differences between sire lines. *p* < 0.05.

**Table 4:** Fertility variables in pigs (mean ± SEM) by cluster of boar ejaculates.

Cluster of Ejaculates	Total Born per Litter	Piglets Born Alive	Stillbirth	Number of Mummies	Litter Weight at Birth (kg)
EC1	9.22 ± 1.21 ^a,b^	8.91 ± 1.25 ^a^	0.17 ± 0.09 ^a^	0.08 ± 0.07 ^a^	15.00 ± 1.88 ^a,b^
EC2	10.37 ± 1.28 ^a,b^	9.55 ± 1.28 ^a^	1.33 ± 0.50 ^b^	0.18 ± 0.09 ^a^	15.72 ± 1.78 ^a,b^
EC3	11.50 ± 1.37 ^a^	10.25 ± 1.32 ^a^	1.50 ± 0.61 ^b^	0.32 ± 0.17 ^a^	18.83 ± 1.71 ^a^
EC4	7.64 ± 1.13 ^b^	7.22 ± 1.14 ^a^	0.12 ± 0.07 ^a^	0.19 ± 0.14 ^a^	11.47 ± 2.03 ^b^

SEM: standard error of the mean. ^a–b^ Different letters indicate differences between clusters. *p* < 0.05.

**Table 5:** Porcine fertility variables (mean ± SEM.) by sire genetic line (percentage variation with respect to Pietrain in brackets).

Sire Line	Total Born per Litter	Piglets Born Alive	Stillbirth	Number of Mummies	Litter Weight at Birth (kg)
Pietrain	8.93 ± 0.93	8.74 ± 0.97	0.28 ± 0.11 ^b^	0.13 ± 0.09	13.88 ± 1.45
Duroc × Pietrain	10.27 ± 0.65 (115.01%)	9.08 ± 0.61 (103.89%)	0.74 ± 0.16 ^a^ (264.29%)	0.24 ± 0.07 (184.61%)	15.58 ± 0.90 (112.2%)

SEM: standard error of the mean. ^a–b^ Different letters indicate differences between sire lines. *p* <0.05.

**Table 6:** Cut-off values of kinematic sperm variables significantly related to litter size fertility, calculated from receiver operating characteristic (ROC) curves.

Variable	Cut-Off Value	Sensitivity (%)	Specificity (%)	Area ROC	*p*-Value
**Total Born per Litter**					
VCL	71.30	56.38	52.58	0.56	0.06
ALH	2.64	50.00	53.33	0.57	0.04
BCF	8.56	55.32	51.55	0.55	0.12
**Piglets Born Alive**					
VCL	71.50	53.19	55.67	0.56	0.06
ALH	2.64	53.57	50.47	0.58	0.03
**Number of Mummies**					
VCL	70.70	56.38	52.58	0.57	0.08
VAP	42.40	54.35	50.51	0.57	0.09
ALH	2.59	50.00	53.33	0.57	0.07

VCL: curvilinear velocity (µm·s^−1^); VAP: average path velocity (µm·s^−1^); ALH: amplitude of lateral head displacement (µm); BCF: beat-cross frequency (Hz).

## Data Availability

The data presented in this study are available within the article.
